# Anti-PLA2R1 Antibodies Containing Sera Induce In Vitro Cytotoxicity Mediated by Complement Activation

**DOI:** 10.1155/2019/1324804

**Published:** 2019-12-30

**Authors:** Maël Lateb, Hajar Ouahmi, Christine Payré, Vesna Brglez, Kevin Zorzi, Guillaume Dolla, Mohamad Zaidan, Sonia Boyer-Suavet, Bertrand Knebelmann, Thomas Crépin, Cécile Courivaud, Noémie Jourde-Chiche, Vincent Esnault, Gérard Lambeau, Barbara Seitz-Polski

**Affiliations:** ^1^Université Côte d'Azur, Centre National de la Recherche Scientifique, Institut de Pharmacologie Moléculaire et Cellulaire, UMR7275, Valbonne Sophia Antipolis, France; ^2^Service de Néphrologie-Dialyse-Transplantation, Hôpital Pasteur, CHU de Nice, Université de Nice-Sophia Antipolis, France; ^3^Centre de Référence Maladies Rares Syndrome Néphrotique Idiopathique, CHU de Nice, Université de Nice-Sophia Antipolis, France; ^4^Service de Néphrologie-Transplantation, Hôpital de Bicêtre, AP-HM France, Université Paris-Saclay, Villejuif, Paris, France; ^5^Université Paris Descartes, Sorbonne Paris Cité, Hôpital Necker-Enfants Malades, Paris, France; ^6^INSERM U1151, Institut Necker Enfants Malades, Hôpital Necker-Enfants Malades, Paris, France; ^7^Département de Néphrologie, Dialyse et Transplantation, Université de Franche-Comté, Besançon, France; ^8^Aix-Marseille Univ, C2VN, INSERM 1263, INRA 1260, AP-HM Hôpital de la Conception, Centre de Néphrologie et Transplantation Rénale, Marseille, France; ^9^Laboratoire d'Immunologie, Hôpital l'Archet, CHU de Nice, Université de Nice-Sophia Antipolis, France

## Abstract

The phospholipase A2 receptor (PLA2R1) is the major autoantigen in idiopathic membranous nephropathy (MN). However, the pathogenic role of anti-PLA2R1 autoantibodies is unclear. Our aim was to evaluate the in vitro cytotoxicity of anti-PLA2R1 antibodies mediated by complement. Forty-eight patients with PLA2R1-related MN from the prospective cohort SOURIS were included. Anti-PLA2R1 titer, epitope profile, and anti-PLA2R1 IgG subclasses were characterized by ELISA. Cell cytotoxicity was evaluated by immunofluorescence in HEK293 cells overexpressing PLA2R1 incubated with patient or healthy donor sera in the presence or absence of rabbit complement or complement inhibitors. Mean cytotoxicity of anti-PLA2R1 sera for HEK293 cells overexpressing PLA2R1 was 2 ± 2%, which increased to 24 ± 6% after addition of rabbit complement (*p* < 0.001) (*n* = 48). GVB-EDTA, which inhibits all complement activation pathways, completely blocked cell cytotoxicity, whereas Mg-EGTA, which only inhibits the classical and lectin pathways, highly decreased suggesting a limited role of the alternative pathway. A higher diversity of IgG subclasses beyond IgG4 and high titer of total IgG anti-PLA2R1 were associated with increased cytotoxicity (*p* = 0.01 and *p* = 0.03 respectively). In a cohort of 37 patients treated with rituximab, high level of complement-mediated cytotoxicity was associated with less and delayed remission at month 6 after rituximab therapy (5/12 vs. 20/25 (*p* = 0.03) in 8.5 months ± 4.4 vs. 4.8 ± 4.0 (*p* = 0.02)). Kaplan-Meier analysis demonstrated that high level of cytotoxicity (≥40%) (*p* = 0.005), epitope spreading (defined by immunization beyond the immunodominant CysR domain) (*p* = 0.002), and high titer of anti-PLA2R1 total IgG (*p* = 0.01) were factors of poor renal prognosis. Anti-PLA2R1 antibodies containing sera can induce in vitro cytotoxicity mediated by complement activation, and the level of cytotoxicity increases with the diversity and the titer of anti-PLA2R1 IgG subclasses. These patients with high level of complement-mediated cytotoxicity could benefit from adjuvant therapy using complement inhibitor associated with rituximab to induce earlier remission and less podocyte injury.

## 1. Introduction

Membranous nephropathy (MN) is an autoimmune disease and a major cause of nephrotic syndrome in adults [1]. It is defined by the presence of subepithelial immune complex deposits with alteration of the glomerular membrane and podocyte injury [2]. Most MN cases are associated with autoantibodies directed against podocyte antigens such as the M-type phospholipase A2 receptor (PLA2R1) [3] and thrombospondin type 1 domain-containing 7A (THSD7A) [4, 5] in 70% and 3% of adult patients, respectively. Disease evolution is highly variable from spontaneous remission to persistent proteinuria or end-stage renal disease [6]. Treatment remains controversial [7, 8]. Kidney Disease Improving Global Outcomes (KDIGO) guidelines recommend a supportive symptomatic treatment (Renine angiotensine system blockers and diuretics) in all patients and immunosuppressive therapy only in the case of persistent nephrotic syndrome or renal function deterioration [9]. Therefore, immunosuppressive treatments are often started only after significant and potentially irreversible complications. New KDIGO guidelines will probably modify this recommendation using new markers to start immunosuppressive therapy [10].

While the pathogenic role of anti-THSD7A antibodies has been proven by the formation of immune deposits and the onset of proteinuria in mice injected with human anti-THSD7A antibodies [11], no such study has been performed for PLA2R1 due to the absence of PLA2R1 expression in mouse or rat podocytes. Nevertheless, PLA2R1 antibody titers rise during clinical activity and decrease before remission [12], and MN recurrence after kidney graft is associated with high titers [13]. Anti-PLA2R1 titers could also predict outcome after immunosuppressive treatment in MN [14]. PLA2R1 epitopes have been identified in three domains of the protein (CysR, CTLD1, and CTLD7), and a mechanism of epitope spreading from the immunodominant CysR domain to CTLD1 and/or CTLD7 domains has been associated with poor prognosis [15–17] corresponding to later stages of the disease [18].

The complement system forms an important part of the innate immune system. It is involved in host defense, but also in autoimmune diseases, and is made up of over 30 proteins that can be sequentially activated in a complex enzymatic cascade. Three major pathways of complement have been described: classical, alternative, and lectin pathways, which are activated by different stimuli [19]. All three pathways converge on complement C3. Cleavage of C3 and C5 successively leads to the production of the membrane attack complex (C5b-9), which is the final common effector [20]. C5b-9 has been shown to have a major role in the Heymann nephritis rat model of MN [21, 22]. The role of the different complement pathways upstream of C5 activation in human and experimental MN remains largely unknown. Primary MN associated to PLA2R1 and THSD7A is characterized by predominant IgG4 (in deposits and in serum) and low amounts of IgG1 and IgG3 [23–25]. Immune complexes typically bind C1q and activate the classical pathway. However, IgG4 does not bind C1q and is considered to be unable to activate the classical pathway [26, 27]. The presence of IgG1 in early deposits could possibly activate the classical pathway, but C1q deposits are very weak in MN [28]. This suggests that the alternative or lectin pathways might be involved in complement activation. Hayashi et al. detected glomerular deposits of C4d, C3d, and C5b-9 in all patients while mannose-binding lectin (MBL) and C1q were detected in only 43% and 46% of patients, respectively [29]. One or more complement pathways may be activated after the formation of immune deposits and can vary among MN patients. Hayashi et al. also described that MN associated with glomerular MBL deposits is more severe. On the other hand, Bally et al. reported cases of PLA2R1-associated MN in patients having a complete MBL deficiency with complement activation mainly due to the alternative pathway [30].

The aim of this study was (i) to evaluate the pathogenic role of anti-PLA2R1 antibodies and the contribution of complement in a simple but routinely used *in vitro* cellular assay using HEK293 cells overexpressing PLA2R1, (ii) identify factors associated with complement mediated-cytotoxicity, and (iii) analyze prognosis value of this complement mediated-cytotoxicity. This study may provide the molecular basis to better understand MN pathogenesis and develop alternative therapeutic strategies.

## 2. Materials and Methods

### 2.1. Patients

Forty-eight patients with PLA2R1-associated MN from the prospective cohort SOURIS were included (NCT02199145) approved by our local Ethic committee. Patients from the SOURIS cohort are primary MN patients (defined by the absence of antinuclear antibodies, negative hepatitis B and C serologies, and negative cancer workup), adults, with MDRD > 30 ml/mn/1.73 m^2^ and anti-PLA2R1 antibodies at diagnosis. The aim of the SOURIS cohort is to demonstrate that epitope profile at diagnosis could guide therapeutic strategy. Patients were enrolled from four French centers from 2015 to 2018 and had a median follow-up of 14 months. Baseline characteristics and follow-up data were recorded until 18 months. Serum anti-PLA2R1 levels, serum creatinine, serum albumin, and proteinuria were measured every 3 months for 18 months. Sera from 20 healthy donors, age- and gender-matched, were collected. Information and written consent were obtained from all patients.

Serum and morning spot urine samples were prospectively collected at the first infusion and every 3 months after the first rituximab infusions (i.e., month 3, month 6). Remissions were defined according to the 2012 KDIGO guidelines. Complete remission was defined by a urinary protein/creatinine ratio < 0.3 g/g accompanied by a normal serum albumin concentration and a preserved kidney function. Partial remission was defined by urinary protein/creatinine ratio < 3.5 g/g with over 50% reduction of proteinuria accompanied by an improvement or normalization of the serum albumin concentration and preserved kidney function.

### 2.2. Anti-PLA2R1 ELISA

The entire extracellular domain of human PLA2R1 or its CysR, CTLD1, and CTLD7 domains bearing a C-terminal HA tag were produced as secreted proteins as described previously [15]. Briefly, pcDNA3.1 expression vectors coding for each protein were transfected into HEK293 cells by calcium phosphate transfection. Three days after transfection, cell medium containing the secreted proteins was collected and centrifuged to remove cell debris. Anti-HA antibodies (Sigma-Aldrich) were coated for one night at 4°C. Plates were then washed three times with PBS/0.02% Tween 20, then blocked for two hours with Seramun Block (Seramun Diagnostica) and then washed again three times. Cell medium from HEK293 cells transfected with the soluble forms of the three PLA2R1 domains or the entire PLA2R1 tagged with HA was then added and incubated for one hour at room temperature on a plate shaker, then washed three times. Patients' sera were diluted at 1 : 100 in PBS/0.1% milk and added to the ELISA plates for two hours. After three washes, anti-human IgG4 (1 : 7500; Southern Biotech) or anti-human IgG1, IgG2, and IgG3, all conjugated with horseradish peroxidase (1 : 5000; Southern Biotech) and diluted in SeramunStab ST plus (Seramun Diagnostica), were incubated for one hour at room temperature on a plate shaker. After three washes, tetramethylbenzidine was added and the reaction was stopped with HCl 1.2 N after 15 minutes. Plates were read at 450 nm. A negative control as well as a highly positive index patient serum was used in each plate to generate a standard curve. The cut-off was optimized by receiver operating characteristic (ROC) curve analysis using 20 healthy controls.

Epitope spreading was defined by anti-CysR reactivity with additional anti-CTLD1 and/or anti-CTLD7 activities.

### 2.3. Measurement of Anti-PLA2R1 Antibodies by ELISA

Serum levels of total IgG anti-PLA2R1 antibodies were measured by the ELISA test developed by EUROIMMUN AG (Lübeck, Germany) [31]. Participants were considered as anti-PLA2R1-positive when levels were higher than 14 RU/ml.

### 2.4. Immunofluorescence Cytotoxicity Assay and Complement Activation Pathway

HEK293 cells overexpressing PLA2R1 with a Tetracycline-Regulated Expression System (PLA2R1/HEK293 T-REx) were obtained as follows. Full-length membrane-bound human PLA2R1 bearing a C-terminal cytoplasmic HA tag was inserted into the pcDNA4/TO expression vector (InvitroGen) and transfected into HEK293 T-REx cells (cultured in DMEM, 10% fetal calf serum with blasticidin 5 *μ*g/ml) using the calcium-phosphate procedure (InvitroGen). One day after transfection, stably expressing cells were selected with zeocine (0.8 mg/ml) for 3 weeks and PLA2R1 expression was induced with tetracycline (1 *μ*g/ml) and validated by western blot as described [15] for 48 hours. Cells were collected by gentle dissociation with trypsin, centrifuged for 3 minutes at 600 rpm, and diluted in OptiMEM to obtain a concentration of 3 × 10^6^ cells/ml.

Cell cytotoxicity was measured as described by Terasaki and McClelland [32]. Three microliters of serum from anti-PLA2R1-positive patients or healthy donors were incubated with 3 × 10^3^ of either induced or noninduced PLA2R1/HEK293 T-REx cells (added in 1 *μ*l) for 1 hour at room temperature in 60-well Terasaki plates (Dutcher©, Strasbourg, France) in duplicates. Cytotoxicity was measured with serial serum dilutions (1 : 1, 1 : 3, and 1 : 10). Serum dilutions were associated to decreased (dilution 1 : 3) or negative (dilution 1 : 10) cytotoxicity (Figure [Supplementary-material supplementary-material-1]). Standard rabbit complement (5 *μ*l/well, Cedarlane©, Ontario, Canada) or OptiMEM was added and incubated for 1 hour at room temperature. Dead cells were revealed after adding 2.5 *μ*l/well of Fluoroquench AO/EB staining/quench (Ingen©Chilly-Mazarin, France) for 10 minutes in the dark. The percentage of dead cells was estimated using a fluorescent microscope (Videomicroscope Zeiss LSM780). Two investigators read plates blindly with a good correlation (rs = 0.81, *p* < 0.0001) and an excellent concordance (ICC = 0.89 (0.85; 0.92)) (Figure [Supplementary-material supplementary-material-1]).

Various versions of gelatin veronal buffer (GVB) were used to determine the complement pathway involved in anti-PLA2R1 cytotoxicity. GVB supplemented with EDTA (GVB-EDTA) (ComplementTech©) was used to inhibit all complement pathways activation, whereas GVB supplemented with magnesium and EGTA (Mg-EGTA) (ComplementTech©) was used to inhibit the classical and lectin pathways, but not the alternative one. Three microliters of three PLA2R1-positive sera were diluted in 5 *μ*l of GVB supplemented with GVB-EDTA or Mg-EGTA and 5 *μ*l of standard rabbit complement. Cytotoxicity was assessed as described above. We confirmed in these conditions the inhibition of the classical pathway activity by measuring CH50 (Total Haemolytic Complement Kits Binding Site©) (Figure [Supplementary-material supplementary-material-1]).

All cytotoxicity assays were performed using the same batch of HEK293 T-Rex cells. A minimum of 50 cells per well was necessary for reading.

### 2.5. Western Blot Analysis

The expression of PLA2R1 in HEK293 cells overexpressing PLA2R1 with a Tetracycline-Regulated Expression System (PLA2R1/HEK293 T-REx) after induction with tetracycline was analyzed by SDS-PAGE under nonreducing conditions. A control without induction with tetracycline was performed. Total proteins (10-50 *μ*g/well) were run on 4-15% precast TGX SDS-PAGE gels (Bio-Rad) and transferred to methanol-soaked PVDF membranes (Bio-Rad) under semidry conditions using Trans-blot Turbo (Bio-Rad) at 25 V constant for 12 min. Membranes were blocked overnight at 4°C in 5% milk with PBS-Tween (PBS-T) 0.05% and then incubated with primary and secondary antibodies for 2 h at room temperature. Primary antibodies were diluted with 0.5% dry milk in PBS-T. Membranes were prepared in replicates and probed with a serum of patient with anti-PLA2R1 antibodies diluted at 1 : 100. Secondary antibody for iMN sera was HRP-conjugated mouse anti-human IgG (Southern Biotech #9200-05) diluted 1 : 30,000 in PBS-T. Membranes were washed three times for 5 min in PBS-T after incubation with primary and secondary antibodies. Detection of protein bands was performed with a chemiluminescent substrate (Millipore) and a Fuji LAS3000 imager.

### 2.6. Statistical Analyses

Data are presented as mean ± SD (for variables with normal distribution) or median and interquartile range (for variables with nonnormal distribution). We used the Shapiro-Wilk test to determine if a variable has a Gaussian distribution. Wilcoxon-Mann–Whitney test and Kruskal–Wallis test were used for comparison between groups. ROC curve analysis was used to define the threshold of each test. Survival curves for remission were calculated using Kaplan–Meier estimates for survival distribution. Correlation between the two readers was assessed by Spearman rank correlation coefficient. Concordance between the two readers was assessed by intraclass correlation coefficient (ICC should be over 0.80 for an excellent concordance). Adjusted analysis was performed using logistic regression. All statistics were performed using the Prism and SAS software. *p* values < 0.05 were considered as statistically significant.

## 3. Results

### 3.1. Patients and Anti-PLA2R1 Antibodies

**Figure 1:**
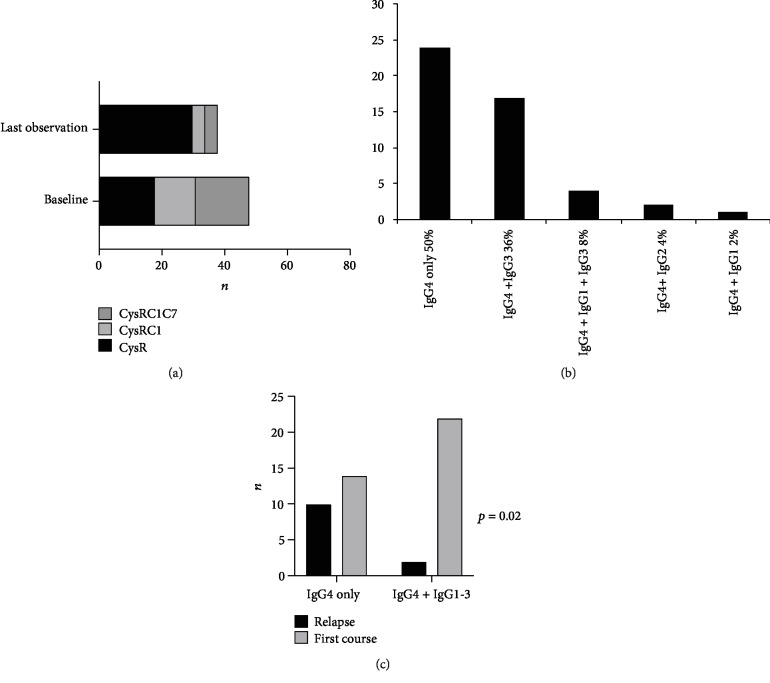
Description of samples with anti-PLA2R1 antibodies tested. (a) Epitope spreading profiles of patients at baseline (*n* = 48) and at last observation (*n* = 36). Follow-up was missing for 12 patients. C1: CTLD1; C7: CTLD7. CysR: immunized against CysR domain alone; CysRC1: immunized against CysR and CTLD1 domains; CysRC1C7: immunized against CysR, CTLD1, and CTLD7 domains. (b) IgG subclass profiles of patients at baseline (*n* = 48). (c) IgG subclass profiles of patients according to disease course: first course or relapse (*n* = 48). Note that patients with the first course of MN have a larger diversity of IgG subclasses (*p* = 0.02).

A total of 48 PLA2R1-positive patients were enrolled in this study (Table 1). Thirty-six patients were included for a first course of MN and 12 patients for a relapse. All patients received conservative therapy with angiotensin-converting-enzyme (ACE) inhibitors and/or angiotensin II receptor blockers. During follow-up, 37 patients were treated with rituximab 1 g at 2-week interval. All patients were positive for the CysR domain of PLA2R1 at baseline, while 30 out of 48 patients (62.5%) had additional antibodies targeting CTLD1 and/or CTLD7 domains (epitope spreading) (Figure 1(a)). At last observation carried forward, epitope spreading was only identified in 6 out of 37 (16.2%) patients. At baseline, all patients had IgG4 anti-PLA2R1, while 24 patients (50%) also had IgG1, IgG2, or IgG3 autoantibodies. Anti-PLA2R1 IgG3 antibodies were the second most represented subclass with 22 (44%) positive patients (Figure 1(b)). Moreover, IgG1, IgG2, IgG3, and IgG4 anti-PLA2R1 antibodies were found in most samples at the first course of the disease, while at relapse, IgG4 anti-PLA2R1 was the predominant subclass (*p* = 0.02) (Figure 1(c)).

### 3.2. Cytotoxicity of Anti-PLA2R1 Autoantibodies Is Dependent on Complement Activation

We choose to test complement-mediated cytotoxicity with a simple, validated, and routinely used test in renal transplantation (the cross-match test), using HEK293 cells overexpressing PLA2R1 with a Tetracycline-Regulated Expression System (instead of lymphocytes in cross-match test).

**Figure 2:**
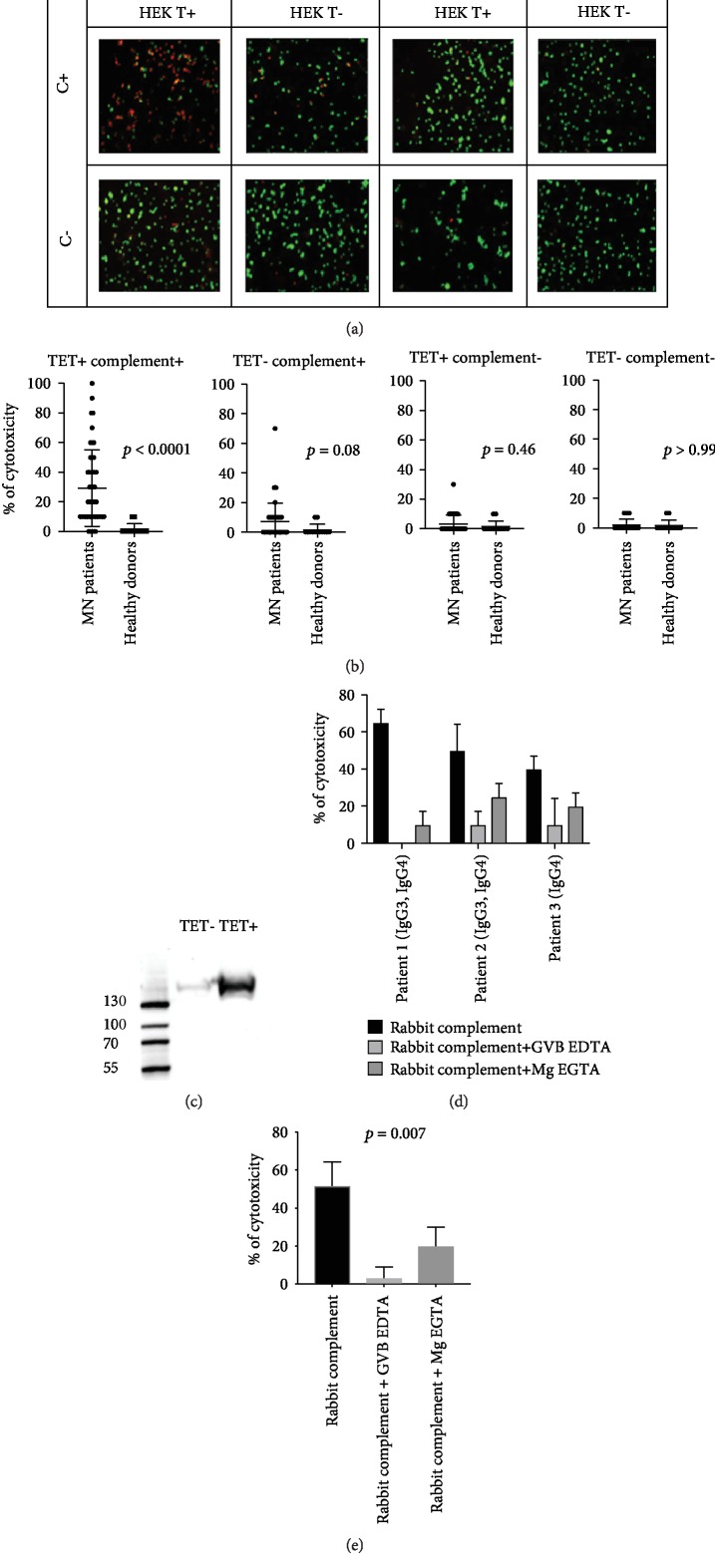
Anti-PLA2R1-mediated cytotoxicity depends on complement in immunofluorescence cytotoxicity assay. (a) Anti-PLA2R1-mediated cytotoxicity depending on complement for a PLA2R1-positive serum and a healthy donor serum. HEK T+: HEK293T-REx cells transfected with PLA2R1 and induced with tetracycline to overexpress PLA2R1. HEK T-: HEK293T-REx cells transfected with PLA2R1 but not induced with tetracycline. C+: addition of rabbit complement. C-: no addition of rabbit complement. Dead cells appear in red; living cells appear in green. (b) Anti-PLA2R1 complement-mediated cytotoxicity in a cohort of 48 patients and 20 healthy donors in different conditions. Note a low level of cytotoxicity in noninduced HEK that tended to be significant (*p* = 0.08) caused by a minimal expression of PLA2R1 even in noninduced cells, as determined by western blot (c). All samples were tested using the same batch of HEK293 cells. A minimum of 50 cells per well was necessary for reading. (c) Expression of PLA2R1 in noninduced and induced HEK293 cells with tetracycline. Note a minimal expression of PLA2R1 in noninduced cells. (d) Analysis of complement activation pathways involved in anti-PLA2R1-mediated cytotoxicity for 3 PLA2R1-positive patients using two inhibitors of complement pathways. Patients 1 and 2 were positive for both IgG3 and IgG4 anti-PLA2R1 antibodies, while patient 3 was only positive for IgG4 anti-PLA2R1. An excess of GVB-EDTA (inhibitor of the three pathways of the complement) or Mg-EGTA (inhibitor of the classical and lectin pathways) was added in serum + complement and the complement-mediated cytotoxicity was measured. All samples were tested using the same batch of HEK293 cells. A minimum of 50 cells per well was necessary for reading. (e) Analysis of complement activation pathways involved in anti-PLA2R1-mediated cytotoxicity for 3 PLA2R1-positive patients. Note that GVB-EDTA strongly inhibits anti-PLA2R1-mediated cytotoxicity in all 3 patients, while Mg-EGTA, which is known to inhibit only the classical and lectin pathways, inhibits only partially the anti-PLA2R1-mediated cytotoxicity, suggesting a potential activation of the alternative pathway in some serum samples. A minimum of 50 cells per well was necessary for reading. HEK293 T-REx cells transfected with PLA2R1 and induced with tetracycline to express PLA2R1 were used. Negative controls (noninduced HEK T-REx cells) are not shown. All cytotoxicity assays were performed using the same batch of HEK293 T-Rex cells. A minimum of 50 cells per well was necessary for reading.

Addition of anti-PLA2R1 serum and rabbit complement to HEK293 cells overexpressing PLA2R1 after induction with tetracycline (PLA2R1/HEK293 T-REx^+^) led to strong cytotoxicity, while all other conditions were barely cytotoxic (Figures 2(a) and 2(b)). In PLA2R1/HEK293 T-REx^+^, the mean cytotoxicity without complement was 2 ± 2% while it increased to 24 ± 6% with complement (*p* < 0.001). In HEK293 cells not induced with tetracycline (PLA2R1/HEK293 T-REx^−^), the mean cytotoxicity was 7 ± 2% and 2 ± 1% with and without complement, respectively. Addition of healthy donor serum was barely cytotoxic, no matter the conditions. The decreased cell viability of PLA2R1/HEK293 T-REx^−^ in the presence of complement was probably caused by a minimal expression of PLA2R1 even in noninduced cells, as determined by western blot (Figure 2(c)). Using ROC curve analysis, we identified a threshold > 10% of cytotoxicity associated with a positive test with a sensitivity of 87.5% and specificity of 84.6% (AUC = 0.90 (0.83 to 0.98) *p* < 0.0001) (Figure [Supplementary-material supplementary-material-1]).

### 3.3. Analysis of the Complement Activation Pathways Involved in Cytotoxicity Mediated by Anti-PLA2R1 Autoantibodies

The complement activation pathways involved in anti-PLA2R1 cytotoxicity were studied with three anti-PLA2R1 serum samples showing a robust level of cytotoxicity (Figure 2(d)). The first two patients were positive for both IgG3 and IgG4 anti-PLA2R1 antibodies, while the third patient was only positive for IgG4. For the first patient, the addition of GVB-EDTA totally inhibited anti-PLA2R1-mediated cytotoxicity (from 65 to 0%) as addition of Mg-EGTA (from 65 to 10%). In the presence of Mg-EGTA, we confirmed a complete blockade of the classical pathway (CH50 < 10%, Figure [Supplementary-material supplementary-material-1]), suggesting that the alternative pathway mediates cytotoxicity for the first patient. For the second patient, the addition of GVB-EDTA strongly inhibited anti-PLA2R1-mediated cytotoxicity (from 50 to 10%), while the addition of Mg-EGTA only decreased anti-PLA2R1-mediated cytotoxicity to lower but still detectable levels (from 50% to 25%). For the third patient, the addition of GVB-EDTA strongly inhibited anti-PLA2R1-mediated cytotoxicity (from 40 to 10%) whereas the addition of Mg-EGTA only inhibited anti-PLA2R1-mediated cytotoxicity by 2-fold (from 40 to 20%) (Figure 2(a)).

In conclusion, GVB-EDTA, which is known to inhibit the three pathways, strongly inhibited anti-PLA2R1-mediated cytotoxicity in all three patients, which confirm the role of the complement, while Mg-EGTA, which is known to inhibit only the classical and the lectin pathways, partially inhibited the anti-PLA2R1-mediated cytotoxicity, suggesting a potential limited activation of the alternative pathway in some serum samples (*p* = 0.007) (Figure 2(e)). Because Mg-EGTA induces inhibition of more than half of the complement-mediated cytotoxicity, the activation of the lectin or the classical pathway seems to be predominant.

### 3.4. Factors Associated with Cytotoxicity Induced by Anti-PLA2R1 Autoantibodies and Mediated by Complement

**Figure 3:**
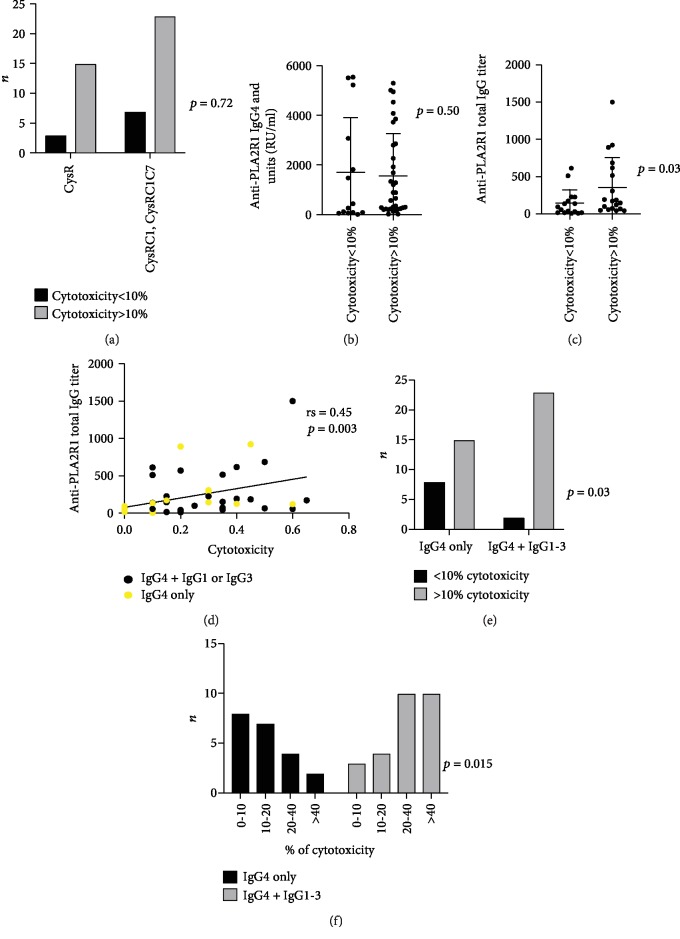
Predictive factors of anti-PLA2R1-mediated cytotoxicity in immunofluorescence cytotoxicity assay. (a) Relationship between anti-PLA2R1-induced cytotoxicity mediated by complement and epitope spreading profile at baseline (*n* = 48). CysR: immunized against CysR domain alone; CysRC1: immunized against CysR and CTLD1 domains; CysRC1C7: immunized against CysR, CTLD1, and CTLD7domains. (b) Relationship between anti-PLA2R1-induced cytotoxicity mediated by complement and Anti-PLA2R1 IgG4 (RU/ml) titers at baseline (*n* = 48). (c) Relationship between anti-PLA2R1-induced cytotoxicity mediated by complement and Anti-PLA2R1 total IgG titer at baseline (*n* = 48). (d) Correlation between Anti-PLA2R1 total IgG titer and complement-mediated cytotoxicity (*n* = 48). (e) Anti-PLA2R1-induced cytotoxicity mediated by complement according to anti-PLA2R1 IgG subclasses at baseline (*n* = 48). (f) Anti-PLA2R1-induced cytotoxicity mediated by complement regrouped by levels of cytotoxicity according to anti-PLA2R1 IgG subclasses at baseline (*n* = 48). Note that patients with high level of cytotoxicity (≥40%) are predominantly IgG4+IgG1-IgG3.

We analyzed the factors that may be associated with cytotoxicity (>10%) among the different serum samples from the 48 patients. Epitope spreading as well as high IgG4 anti-PLA2R1 titers was not associated with decreased cell viability (*p* = 0.72 and *p* = 0.50, respectively) (Figures 3(a) and 3(b)) while high level of total anti-PLA2R1 total IgG titer was associated with cytotoxicity (*p* = 0.03, Figure 3(c)); moreover, these two variables were correlated (rs = 0.45, *p* < 0.001) (Figure 3(d)). As suggested, the presence of anti-PLA2R1 IgG1, IgG2, or IgG3 antibodies was associated with increased cell cytotoxicity (*p* = 0.03) (Figure 3(e)). Median complement-mediated cytotoxicity was significantly higher for serum samples containing different IgG subclasses versus those containing only anti-PLA2R1 IgG4 antibodies (30% (10; 40) versus 10% (0; 30), *p* = 0.02). There was an inverse relationship for complement-mediated cytotoxicity between serum samples containing multiple IgG subclasses and those containing only IgG4, suggesting that IgG subclasses other than IgG4 drive the observed cytotoxic effects (*p* = 0.015) (Figure 3(f)). We failed to identify a correlation between titers of each subclass of IgG and cytotoxicity (Figure [Supplementary-material supplementary-material-1]) suggesting that it is the whole of subclasses that is responsible for the cytotoxic effect.

### 3.5. Prognostic Factors for Remission

During follow-up, 37 patients were treated with rituximab 1 g at 2-week interval, 25 entered into remission at month 6 (67.6%). Time to remission after treatment was 6.1 ± 4.4 months.

We compared clinical characteristics of patients with the highest level of cytotoxicity (the third tertile defined by a threshold of 40% or more of complement-mediated cytotoxicity) (Table 2). Patients with complement-mediated cytotoxicity ≥ 40% were similar at diagnosis for age and sex ratio but have the highest level of proteinuria (6.65 (5.75; 9.86) vs. 4.90 (3.23; 7.68), *p* = 0.03) and total IgG anti-PLA2R1 titer at diagnosis (192 (120; 686) vs. 94 (33; 199), *p* = 0.01) while IgG4 anti-PLA2R1 titer and level of epitope spreading were similar (1840 (322; 3990) vs. 1338 (220; 3461), *p* = 0.62, and 9/12 (75%) vs. 13/25 (52%), *p* = 0.08, respectively). After treatment with rituximab, fewer patients with high level of complement-mediated cytotoxicity entered into remission (5/12 (42%) vs. 20/25 (80%), *p* = 0.03) in longer time (8.5 months ± 4.4 vs. 4.8 ± 4.0, *p* = 0.02) with higher proteinuria at month 6 (3.10 (2.51; 5.60) vs. 1.21 (0.30; 2.32), *p* = 0.001).

**Figure 4:**
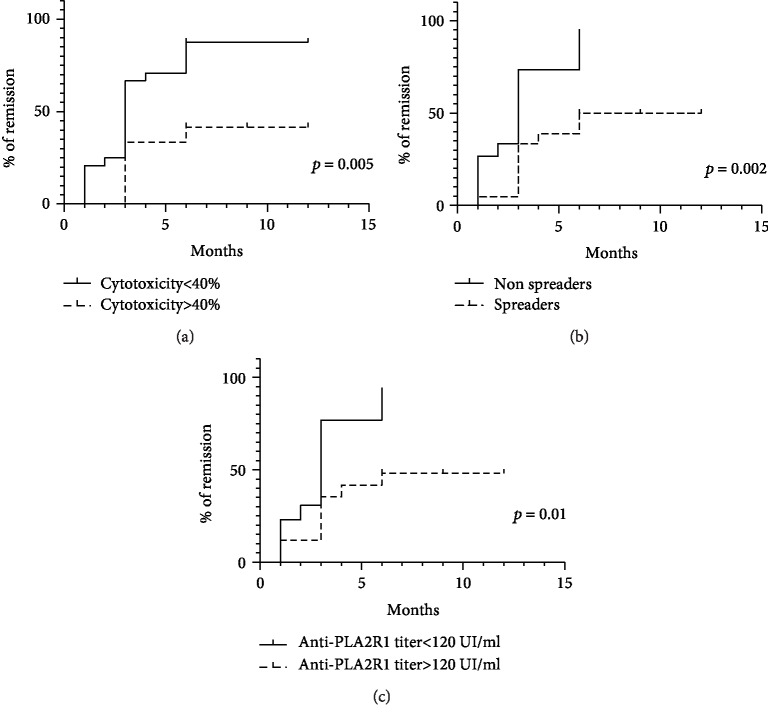
Factors associated with remission. Renal event is defined by remission (partial or complete) one year after diagnosis of MN. (a) Percent of patients achieving remission according to the level of complement-mediated cytotoxicity (*n* = 37). (b) Percent of patients achieving remission according to epitope spreading profile (*n* = 37). (c) Percent of patients achieving remission according to the level of total IgG anti-PLA2R1 (*n* = 37).

We then analyzed factors associated with remission at month 6 (Table 3) (using univariate analysis): high anti-PLA2R1 total IgG titer, epitope spreading, and high level of cytotoxicity (≥40%) were associated with active disease at month 6 after 2 pulses of rituximab (185.0 (147.0; 893.0) vs. 73.0 (39.5; 209.0) (*p* = 0.01), 10/12 (83%) vs. 11/25 (44%) (*p* = 0.04), and 7/12 (58%) vs. 5/25 (20%) (*p* = 0.03), respectively). As all these factors were correlated (Figure 1(c)and Figure [Supplementary-material supplementary-material-1]), multivariate analysis failed to identify an independent prognosis factor. Because each remission occurred at different time points, we performed a time-to-event analysis of renal survival. Renal event was defined by achieving remission within the year after the first course of rituximab. The rate of remission was significantly lower for patients with high level of cytotoxicity (≥40%) (*p* = 0.005, Figure 4(a)), with epitope spreading (*p* = 0.002, Figure 4(b)), and high titer of total IgG anti-PLA2R1 (*p* = 0.01, Figure 4(c)).

Patients with high level of cytotoxicity (≥40%) had six fold decreased chance to achieve remission (Odds ratio = 6.33 (1.39; 28.69)) with a mean time to achieve remission of 8.5 months ± 4.4 months vs. 4.8 months ± 4.0 months (*p* = 0.02).

## 4. Discussion

Our results demonstrate that anti-PLA2R1 antibodies induce in vitro cytotoxicity mediated by complement activation as suggested by Kistler et al. who analyzed TRPC6 protective role from complement-mediated podocyte injury [33]. Samples with high titer of anti-PLA2R1 total IgG have higher level of complement-mediated cytotoxicity, and these patients had exhibited a bad prognosis. In a previous work, we used human podocyte line in our standard conditions of western blot (using 10-50 *μ*g/ml of total protein) to determine the approximate expression of PLA2R1 in human podocytes and observed a signal using positive control MN sample with PLA2R1 antibodies [13] as we observed in similar conditions using HEK293 cells overexpressing PLA2R1. This suggested that the level of PLA2R1 expression in HEK 293 cells overexpressing PLA2R1 is approximately comparable with the level of PLA2R1 expression in human podocyte.

We demonstrate that the level of cytotoxicity correlates with total IgG anti-PLA2R1 titer and not with IgG4 anti-PLA2R1. Patients with high level of cytotoxicity have more active disease at diagnosis, lower chance of remission after rituximab therapy, and longer time to enter into remission. Complement inhibitor (GVB-EDTA) strongly inhibited this cytotoxicity. If our results are confirmed in other studies, MN patients with high level of anti-PLA2R1 antibodies and complement-mediated cytotoxicity could benefit from adjuvant therapy using complement inhibitor associated with rituximab, which could probably induce less podocyte injury and earlier remissions.

There is a large body of evidence that anti-PLA2R1 IgG4 is predominant in MN [31, 34]. All patients were positive for IgG4 anti-PLA2R1. However, in addition to anti-PLA2R1 IgG4 antibodies, we observed in some patients the presence of IgG1, IgG2, or IgG3 anti-PLA2R1, as previously described [35, 36]. Moreover, IgG4 was the predominant subclass in relapsing MN. This result is in accordance with the IgG subclass switch from IgG1, IgG2, and IgG3 to IgG4 observed in renal biopsies during disease progression by Huang et al. [25]. A similar IgG subclass switch was observed in other IgG4-mediated autoimmune diseases such as pemphigus vulgaris and idiopathic thrombotic thrombocytopenic purpura [25, 37, 38].

The exact contribution of each of the three complement pathways in MN remains unknown. The lectin pathway may play an important role, since the prevalence and staining intensity of mannose-binding lectin (MBL) deposits were much higher in PLA2R1-positive patients than in patients without MBL deposits [29]. The staining intensity of MBL in glomeruli also correlated with the IgG4 staining intensity and was an unfavorable predictor for remission of proteinuria and renal dysfunction. It is unclear how the lectin pathway (that is known to be an antibody-independent pathway) is involved in MN (that is mediated by anti-PLA2R1 antibodies). Some authors hypothesize that abnormalities of galactosylation of IgG anti-PLA2R1 could activate the lectin pathway [29]. We demonstrated that inhibition of the classical and lectin pathways by Mg-EGTA inhibited the majority of complement-mediated cytotoxicity (completely for one patient and partially for two other patients). This partial inhibition would suggest a limited role of the alternative pathway in anti-PLA2R1-mediated cytotoxicity, which is still active in the presence of Mg-EGTA. Classical or lectin pathways are probably the predominant pathways implicated in MN. Bally et al. reported that PLA2R1-associated MN could develop in patients with IgG3 kappa anti-PLA2R1 antibodies having a complete MBL deficiency but capable of residual complement activation mainly due to the alternative pathway [30]. Moreover, Seikrit et al. described a case of rapidly progressive renal failure in a patient with membranous nephropathy, related to the appearance of antibodies against the complement regulatory protein, factor H. Inhibition of factor H led to hyperactivation of the alternative complement pathway [39]. This mechanism could explain the alternative pathway activation in some MN patients.

We described that during the first course of MN, several serum samples contained anti-PLA2R1 IgGs not restricted to the IgG4 subclass and these samples could induce more cytotoxicity than those containing only anti-PLA2R1 IgG4 autoantibodies. Thus, we suggest that in the presence of several anti-PLA2R1 IgG subclasses, several complement pathways may be activated, and their contribution will depend on the respective antibody titers and ratios between the different IgG subclasses. Segawa et al. also demonstrated that the activation of the different complement pathways varied according to the IgG subclasses presented in MN biopsy specimens [40]. It would be interesting to test which complement pathway can be activated by each individual IgG subclass of anti-PLA2R1 antibodies.

In different forms of human MN and animal models mediated by other antigens, each pathway of complement seems to be involved. Vivarelli et al. demonstrated a predominant role of the classical pathway for neutral endopeptidase protein-associated MN [41]. In a mouse model of MN, Luo et al. showed a role of the alternative pathway in the pathogenicity induced by glomerular subepithelial immune complexes [42]. However, no C3 deposition has been found in the renal tissue of mice following immunization with rabbit anti-THSD7A antibodies or purified human anti-THSD7A while these antibodies induced proteinuria and IgG deposits. These results suggest that complement activation is not vital in the initiation of podocyte injury and proteinuria in this model [43, 44].

Based on our data, we can hypothesize a multistep mechanism of anti-PLA2R1 cytotoxicity: at disease onset, sera containing multiple IgG subclasses (including IgG4) induce cytotoxicity mediated by various complement pathways, then anti-PLA2R1 IgG4 which becomes the predominant subclass leads to the inhibition of PLA2R1 interaction with collagen from the glomerular basement membrane [45–47] at this time, complement-mediated cytotoxicity is not the main pathogenic mechanism. A similar scenario has been described in other autoimmune diseases mediated by IgG4, like in idiopathic thrombotic thrombocytopenic purpura, where an IgG4 subclass switching is associated with increased inhibition of ADAMTS13 enzymatic activity by anti-ADAMTS13 IgG4 antibodies [38]. These findings should now be confirmed in vitro with a podocyte model but will be more difficult to confirm in *in vivo* animal models due to the lack of expression of PLA2R1 in the mouse kidney [48, 49].

Our results suggest a potential benefit of the use of eculizumab (a monoclonal anti-C5) in membranous nephropathy. As we demonstrated, all samples were not equivalent in complement mediated-cytotoxicity. This treatment could be beneficial in only a part of MN patients with a large diversity of IgG subclasses, high level anti-PLA2R1 total IgG, and high level of complement-mediated cytotoxicity.

This study has several limitations. First, we could not demonstrate the role of complement in all sera, probably because of the lack of sensitivity of our system but we choose a simple, validated, and routinely used test as performed in cross-match test before kidney transplantation. We cannot exclude other mechanisms of cytotoxicity not mediated by complement. Secondly, we indirectly demonstrated a limited role of the alternative pathway with inhibition assays. Third, we did not study the lectin pathway that would probably play a role in MN, which is an IgG4-related disease [50].

In conclusion, we have made several observations likely relevant to the mechanism of pathogenesis in MN. First, our in vitro data showed that anti-PLA2R1 antibodies induce complement-mediated cytotoxicity. Second, this complement-mediated cytotoxicity is associated with high level of anti-PLA2R1 total IgG and more severe disease. Third, classical and/or lectin pathways are predominant. However, the role of each individual anti-PLA2R1 IgG subclass relative to each of the three complement pathways needs to be addressed in further studies, with a possible sequential scenario where the IgG subclass switch would also be associated with a switch of the complement activation pathways, from the classical and alternative pathways to the MBL pathway, as both the autoimmune response and the disease activity progress. Finally, if our results are confirmed, our findings would suggest that MN patients could benefit not only from targeted therapy on B cells but also from a therapy using complement inhibitors and eventually combining the two therapeutic strategies towards a more effective treatment for some MN patients with high level of cytotoxicity to reduce podocyte injury.

## Figures and Tables

**Table 1:** Clinical characteristics of patients in the study.

Clinical characteristics	Cohort SOURIS (48 patients)
Age (years)	59 ± 15
Gender ratio (M/F)	3.3
LOCF (months)	14 (3; 18)
Proteinuria at baseline (g/g)	5.1 (3.5; 7.2)
Serum albumin at baseline (g/l)	23.9 (21.2; 29.7)
Serum creatinine at baseline (*μ*mol/l)	103 (84.7; 135)
Proteinuria at LOCF (g/g)	1.9 (0.3; 3.4)
Patients treated with rituximab	37
Patients treated with other immunosuppressors (Ponticelli)	4

Data are presented as mean ± SD or as median and interquartile range. LOCF: last observation carried forward.

**Table 2:** Factors associated with cytotoxicity ≥ 40% (*n* = 37 patients treated with rituximab).

	Cytotoxicity ≥ 40% (*n* = 12)	Cytotoxicity < 40% (*n* = 25)	*p* value
Age	60 ± 11	57 ± 16	0.46
Sex ratio (M/F)	10/2	19/6	>0.99
Proteinuria at diagnosis (g/g)	6.65 (5.75; 9.86)	4.90 (3.26; 7.68)	0.03^∗^
Anti-PLA2R1 total IgG (RU/ml) at diagnosis	192 (120; 686)	94 (33; 199)	0.01^∗^
Anti-PLA2R1 IgG4 (RU/ml) at diagnosis	1840 (322; 3990)	1338 (220–3461)	0.62
Spreaders at diagnosis	10/12 (83%)	12/25 (48%)	0.07
Proteinuria at month 6 (g/g)	3.70 (2.51; 5.60)	1.21 (0.30; 2.32)	0.001^∗^
Remission at month 6	5/12 (42%)	20/25 (80%)	0.03^∗^
Time to remission (months)	8.5 ± 4.4	4.8 ± 4.0	0.02^∗^

Data are presented as *n* (%) or mean ± SD or as median with interquartile range. Remission is defined as proteinuria < 3.5 g/g and serum albumin > 30 g/l. Spreaders are defined by samples with anti-CysR reactivity with additional anti-CTLD1 and/or anti-CTLD7 activities additional to anti-CysR reactivity.

**Table 3:** Factors associated with remission at month 6 in patients treated with rituximab (*n* = 37).

	Remission at month 6	No remission at month 6	*p* value
Age	55 ± 15	64 ± 14	0.12
Sex ratio (M/F)	2.3	11	0.21
Proteinuria at diagnosis (g/g)	4.9 (3.3; 10.4)	7.2 (4.6; 11.4)	0.08
Anti-PLA2R1 total IgG (RU/ml) at diagnosis	73.0 (39.5; 209.0)	185.0 (147.0; 893.0)	0.01^∗^
Anti-PLA2R1 IgG4 (RU/ml) at diagnosis	885.0 (220.5; 2669.0)	2824.0 (420.5; 5538.0)	0.12
Spreaders at diagnosis	11/25 (44%)	10/12 (83%)	0.04^∗^
Complement-mediated cytotoxicity ≥ 40%	5/25 (20%)	7/12 (58%)	0.03^∗^

Data are presented as *n* (%) or mean ± SD or as median with interquartile range. Remission is defined as proteinuria < 3.5 g/g and serum albumin > 30 g/l. Spreaders are defined by samples with anti-CysR reactivity with additional anti-CTLD1 and/or anti-CTLD7 activities additional to anti-CysR reactivity.

## Data Availability

The datasets used and/or analyzed during the current study are available from the corresponding author on reasonable request (after ethics approval).

## Abbreviations

MN: Membranous nephropathy
PLA2R1: M-type phospholipase A2 receptor
THSD7A: Thrombospondin type 1 domain-containing 7A
KDIGO: Kidney Disease Improving Global Outcomes
MBL: Mannose-binding lectin.

## Supplementary Materials

Supplementary Materials Figure Sup 1A: serum dilutions were associated to decreased (dilution 1 : 3) or negative (dilution 1 : 10) cytotoxicity. Standard rabbit complement (5 *μ*l/well, Cedarlane©, Ontario, Canada) or OptiMEM was added and incubated for 1 hour at room temperature. Dead cells were revealed after adding 2.5 *μ*l/well of Fluoroquench AO/EB staining/quench (Ingen©Chilly-Mazarin, France) for 10 minutes in the dark. The percentage of dead cells was estimated using a fluorescent microscope (Videomicroscope Zeiss LSM780). Figure Sup 1B and 1C: two investigators read plates blindly with a good correlation (rs = 0.81, *p* < 0.0001) (Figure 1(b)) and an excellent concordance (ICC = 0.89 (0.85; 0.92)) (Figure 1(c)). Figure Sup 2: various versions of gelatin veronal buffer (GVB) were used to determine the complement pathway involved in anti-PLA2R1 cytotoxicity. GVB supplemented with EDTA (GVB-EDTA) (ComplementTech©) was used to inhibit all complement activation pathways, whereas GVB supplemented with magnesium and EGTA (Mg-EGTA) (ComplementTech©) was used to inhibit the classical and lectin pathways, but not the alternative one. Three microliters of three PLA2R1-positive sera were diluted in 5 *μ*l of GVB supplemented with GVB-EDTA or Mg-EGTA and 5 *μ*l of standard rabbit complement. Cytotoxicity was assessed as described above. We confirmed in these conditions the inhibition of the classical pathway activity by measuring CH50 (Total Haemolytic Complement Kits Binding Site©). All cytotoxicity assays were performed using the same batch of HEK293 T-Rex cells. A minimum of 50 cells per well was necessary for reading. Figure Sup 3: ROC curve of cytotoxicity between MN patients and healthy donors. Using ROC curve analysis, we identified a threshold > 10% of cytotoxicity associated with a positive test with a sensitivity at 87.5% and specificity at 84.6% (AUC = 0.90 (0.83 to 0.98), *p* < 0.0001). Figure Sup 4: correlation between anti-PLA2R11 titer and epitope spreading.
