# Supplementary material for: Anti-PLA2R1 Antibodies Containing Sera Induce In Vitro Cytotoxicity Mediated by Complement Activation
Source: J Immunol Res. 2019 Dec 30;2019:1324804. doi: 10.1155/2019/1324804 (PMC7012209; doi:10.1155/2019/1324804)
Supplement: Supplementary Materials — Figure Sup 1A: serum dilutions were associated to decreased (dilution 1 : 3) or negative (dilution 1 : 10) cytotoxicity. Standard rabbit complement (5 μl/well, Cedarlane©, Ontario, Canada) or OptiMEM was added and incubated for 1 hour at room temperature. Dead cells were revealed after adding 2.5 μl/well of Fluoroquench AO/EB staining/quench (Ingen©Chilly-Mazarin, France) for 10 minutes in the dark. The percentage of dead cells was estimated using a fluorescent microscope (Videomicroscope Zeiss LSM780). Figure Sup 1B and 1C: two investigators read plates blindly with a good correlation (rs = 0.81, p < 0.0001) (Figure 1(b)) and an excellent concordance (ICC = 0.89 (0.85; 0.92)) (Figure 1(c)). Figure Sup 2: various versions of gelatin veronal buffer (GVB) were used to determine the complement pathway involved in anti-PLA2R1 cytotoxicity. GVB supplemented with EDTA (GVB-EDTA) (ComplementTech©) was used to inhibit all complement activation pathways, whereas GVB supplemented with magnesium and EGTA (Mg-EGTA) (ComplementTech©) was used to inhibit the classical and lectin pathways, but not the alternative one. Three microliters of three PLA2R1-positive sera were diluted in 5 μl of GVB supplemented with GVB-EDTA or Mg-EGTA and 5 μl of standard rabbit complement. Cytotoxicity was assessed as described above. We confirmed in these conditions the inhibition of the classical pathway activity by measuring CH50 (Total Haemolytic Complement Kits Binding Site©). All cytotoxicity assays were performed using the same batch of HEK293 T-Rex cells. A minimum of 50 cells per well was necessary for reading. Figure Sup 3: ROC curve of cytotoxicity between MN patients and healthy donors. Using ROC curve analysis, we identified a threshold > 10% of cytotoxicity associated with a positive test with a sensitivity at 87.5% and specificity at 84.6% (AUC = 0.90 (0.83 to 0.98), p < 0.0001). Figure Sup 4: correlation between anti-PLA2R11 titer and epitope spreading. [file 1324804.f1.pdf]

Supplementary materials

Figure Sup 1A

Serum dilutions were associated to decrease (dilution 1:3) or negative (dilution 1:10) cytotoxicity. (Figure Sup 1A). Standard rabbit complement (5 µl/well, Cedarlane©, Ontario, Canada) or OptiMEM were added and incubated for 1 hour at room temperature. Dead cells were revealed after adding 2.5 µl/well of Fluoroquench AO/EB staining/quench (Ingen©Chilly-Mazarin, France) for 10 minutes in the dark. The percentage of dead cells was estimated using a fluorescent microscope (Videomicroscope Zeiss LSM780).

Figure sup 1B and 1C

Two investigators read plates blindly with a good correlation (rs=0.81 p<0.0001) (Figure 1B) and an excellent concordance (ICC=0.89 [0.85; 0.92] (Figure 1C).

Figure Sup 2

Various versions of gelatin veronal buffer (GVB) were used to determine the complement pathway involved in anti-PLA2R1 cytotoxicity. GVB supplemented with EDTA (GVB-EDTA) (ComplementTech©) was used to inhibit all complement activation pathways, whereas GVB supplemented with magnesium and EGTA (Mg-EGTA) (ComplementTech©) was used to inhibit the classical and lectin pathways, but not the alternative one. Three microliters of three PLA2R1-positive sera were diluted in 5 µl of GVB supplemented with GVB-EDTA or Mg-EGTA and 5 µl of standard rabbit complement. Cytotoxicity was assessed as described above. We confirmed in these conditions the inhibition of the classical pathway activity by measuring CH50 (Total Haemolytic Complement Kits Binding Site©) (Figure Sup 2).

All cytotoxicity assays were performed using the same batch of HEK293 T-Rex cells. A minimal of 50 cells per well was necessary for reading.

Figure Sup 3. ROC Curve of cell cytotoxicity between MN patients and healthy donors

Using ROC curve analysis, we identified a threshold >10% of cytotoxicity associated with a positive test with a sensitivity at 87.5% and specificity at 84.6% (AUC=0.90 [0.83 to 0.98] $p<0.0001$)

Figure sup 4. Correlation between anti-PLA2R1 IgG1, 2, 3 and 4 titer and complement-mediated cytotoxicity.

OD: Optic Density

Figure sup 5. Correlation between anti-PLA2R1 titer and epitope spreading

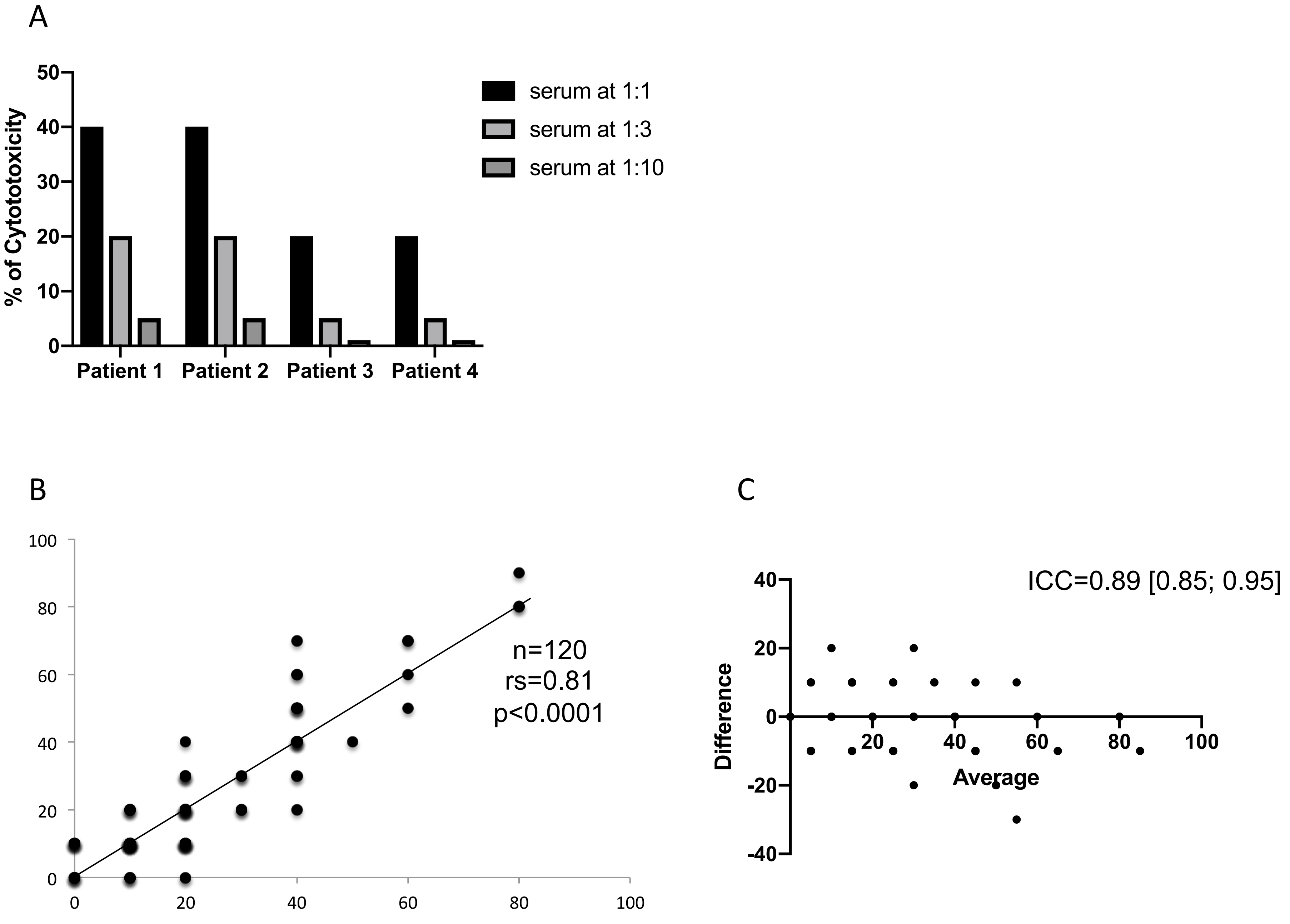
A
50
40
30
20
10
0
% of Cytotoxicity
Patient 1
Patient 2
Patient 3
Patient 4
serum at 1:1
serum at 1:3
serum at 1:10
B
100
80
60
40
20
0
0
20
40
60
80
100
n=120
rs=0.81
p<0.0001
C
ICC=0.89 [0.85; 0.95]
40
20
0
-20
-40
Difference
20
40
60
80
100
Average

Figure Sup 1

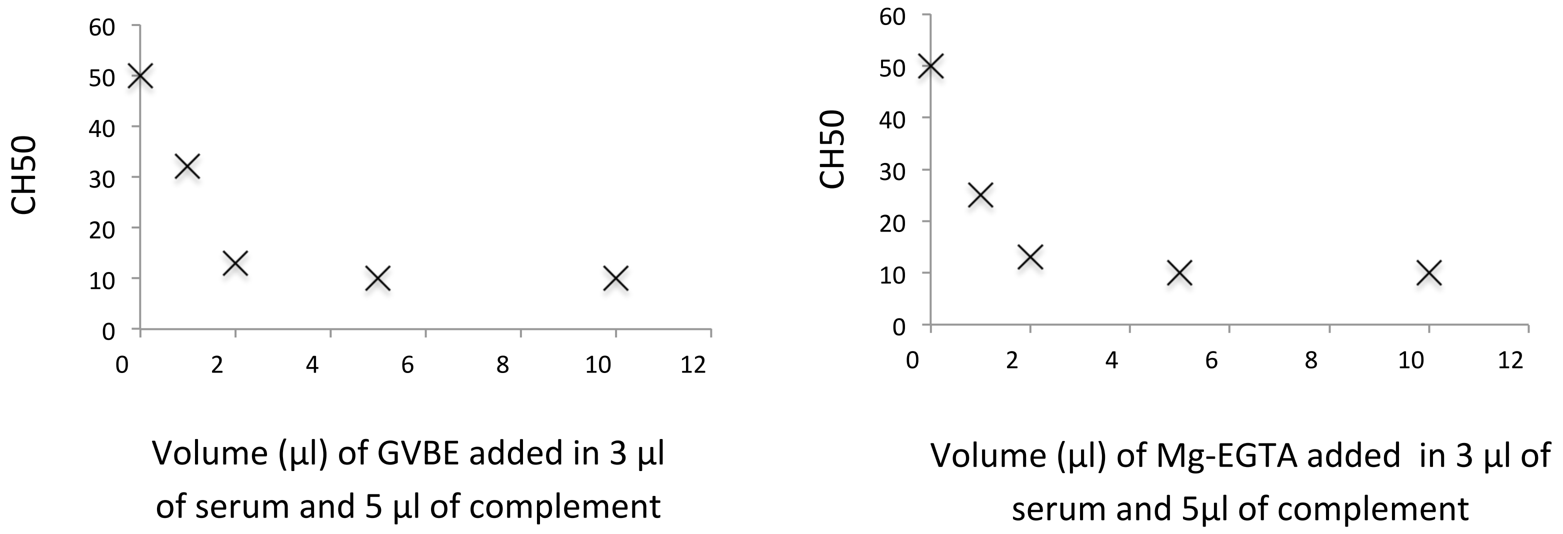
CH50
60
50
40
30
20
10
0
0
2
4
6
8
10
12
Volume (µl) of GVBE added in 3 µl of serum and 5 µl of complement
CH50
60
50
40
30
20
10
0
0
2
4
6
8
10
12
Volume (µl) of Mg-EGTA added in 3 µl of serum and 5µl of complement

Figure Sup 2

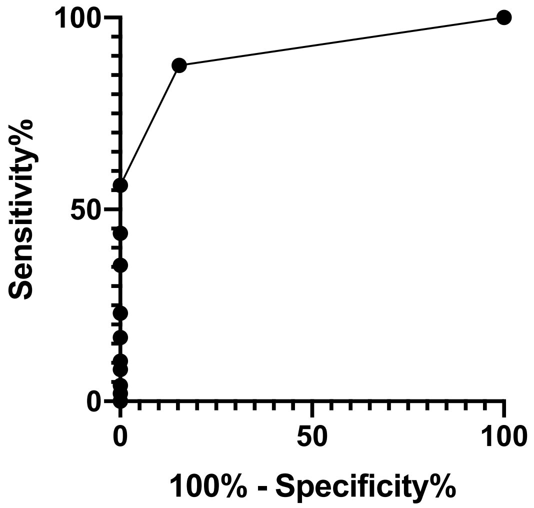

AUC=0.90
p<0.0001
Threeshold<10% Se=87.50 Spe=84.62

Figure Sup 3

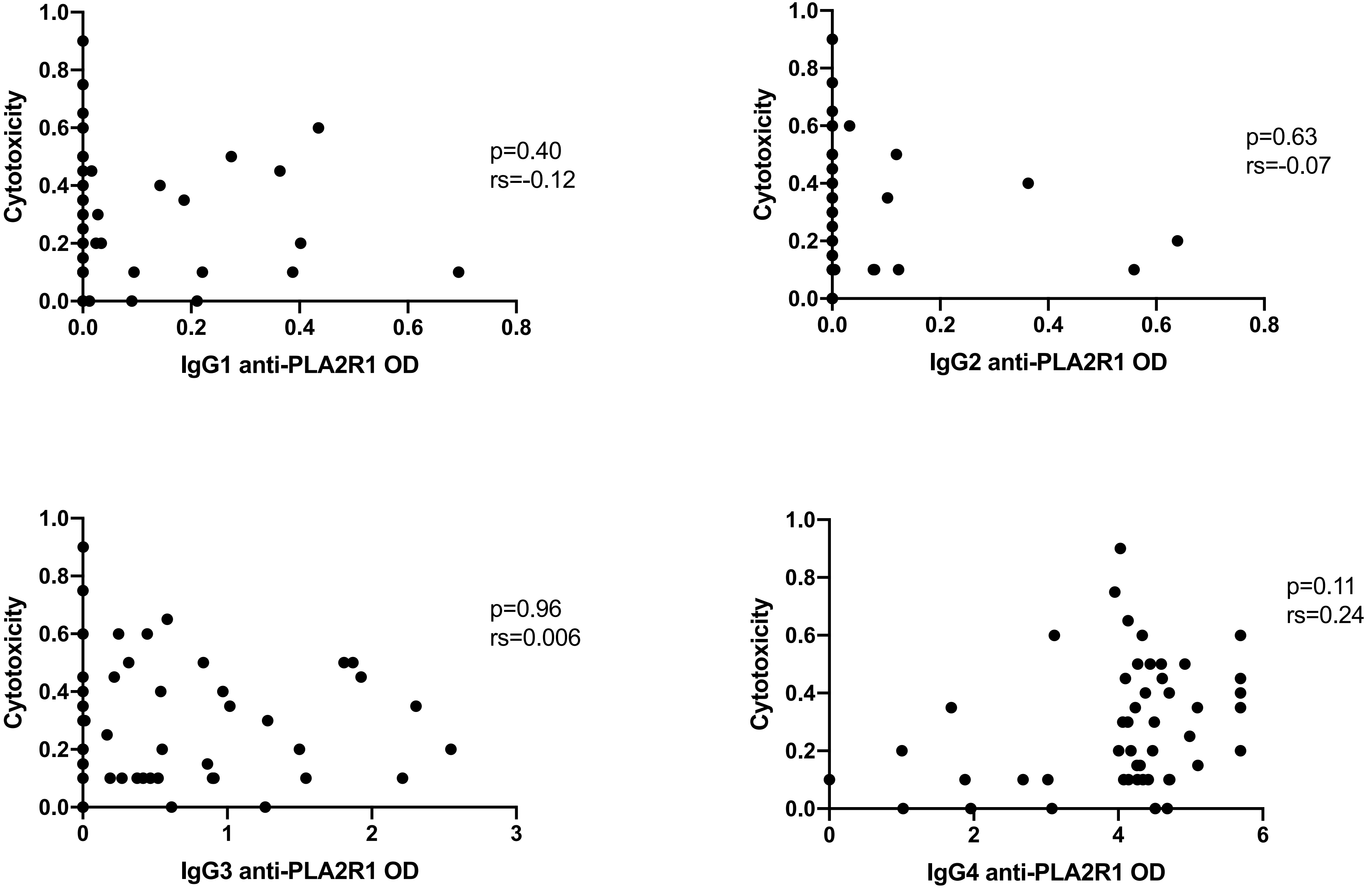
Cytotoxicity
IgG1 anti-PLA2R1 OD
p=0.40
rs=-0.12
Cytotoxicity
IgG2 anti-PLA2R1 OD
p=0.63
rs=-0.07
Cytotoxicity
IgG3 anti-PLA2R1 OD
p=0.96
rs=0.006
Cytotoxicity
IgG4 anti-PLA2R1 OD
p=0.11
rs=0.24

Figure sup 4

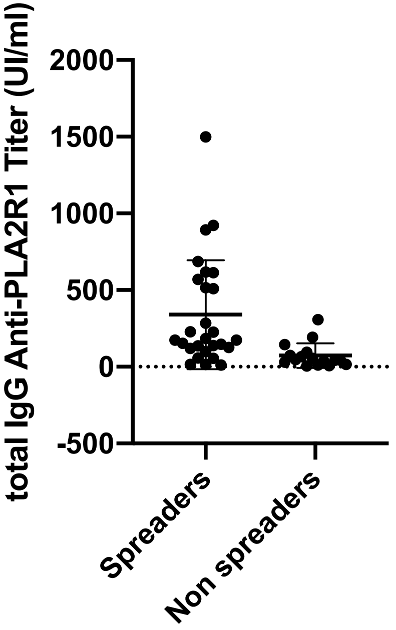

p=0.0004

Figure sup 5
